# Modulation of pathogenic oral biofilms towards health with nisin probiotic

**DOI:** 10.1080/20002297.2020.1809302

**Published:** 2020-08-24

**Authors:** Allan Radaic, Changchang Ye, Brett Parks, Li Gao, Ryutaro Kuraji, Erin Malone, Pachiyappan Kamarajan, Ling Zhan, Yvonne L. Kapila

**Affiliations:** a Department of Orofacial Sciences, School of Dentistry, University of California San Francisco, San Francisco, CA, USA; b State Key Laboratory of Oral Diseases, National Clinical Research Center for Oral Diseases, Department of Periodontology, West China School of Stomatology, Sichuan University, Chengdu, China; c Department of Periodontology, Guanghua School of Stomatology, Hospital of Stomatology, Sun Yat-sen University, Guangzhou, China; d Department of Life Science Dentistry, The Nippon Dental University, Tokyo, Japan; e Department of Periodontology, The Nippon Dental University School of Life Dentistry at Tokyo, Tokyo, Japan

**Keywords:** Nisin, *lactococcus lactis*, oral biofilm, oral health, oral bacteria

## Abstract

**Background:**

Oral dysbiosis is an imbalance in the oral microbiome and is associated with a variety of oral and systemic diseases, including periodontal disease, caries, and head and neck/oral cancer. Although antibiotics can be used to control this dysbiosis, they can lead to adverse side effects and superinfections. Thus, novel strategies have been proposed to address these shortcomings. One strategy is the use of probiotics as antimicrobial agents, since they are considered safe for humans and the environment. Specifically, the Gram-positive *Lactococcus lactis*, a species present in the oral and gut microbiota, is able to produce nisin, which has been used worldwide for food preservation.

**Objective:**

The objective of this study was to test whether a nisin probiotic can promote a healthier oral microbiome in pathogen-spiked oral biofilms.

**Results:**

We found that *L. lactis* can prevent oral biofilm formation and disrupt 24-h and 48-h pre-formed biofilms. Finally, we demonstrate that both treatments, a nisin-producing *L. lactis* probiotic and nisin can decrease the levels of pathogens in the biofilms and return the diversity levels back to control or ‘healthy’ levels.

**Conclusion:**

A nisin-producing probiotic, can be used to treat ‘disease-altered’ biofilms and promote healthier oral biofilms, which may be useful for improving patient oral health.

## Introduction

The human microbiome lives in a symbiotic or mutualistic relationship with the human host and assists with the development of the host defense system, regulation of the gastrointestinal and cardiovascular system, nutrient absorption and energy regulation [1–3]. The oral microbiome is comprised of hundreds of microorganisms that form multispecies oral biofilms [3,4]. Oral biofilms play an essential role both in the development of the natural oral physiology and defense of the host [5].

Because of these important roles, an imbalance in the oral microbiome or development of dysbiosis is associated with a variety of oral and systemic diseases, including periodontal disease, caries, recurrent endodontic infections, and head and neck cancer (HNC) [6,7]. Although antibiotics can be used to control this dysbiosis, they can lead to superinfections. Thus, novel strategies need to be proposed to address this shortcoming. One of these strategies is the use of bacteriocins and probiotics to assist in mitigating this dysbiosis by suppressing oral pathogens within these communities.

Recently, the potential for using a nisin bacteriocin and nisin probiotic in biomedical applications has been highlighted [8–11]. Nisin is a class I Lantibiotic bacteriocin produced by the Gram-positive bacterium *Lactococcus lactis* and it contains 34 amino acids in a penta-cyclic structure [12,13]. Nisin is active against both Gram-positive and Gram-negative bacteria, including *Streptococcus aureus, Listeria monocytogenes, Fusobacterium nucleatum, Porphyromonas gingivalis* and *Treponema denticola* [8,12]. Nisin itself and nisin-expressing *L. lactis* spp. have been used successfully to abrogate infections associated with drug-resistant pathogens, gastrointestinal infections, respiratory tract infections, skin and soft tissue infections, mastitis, HNC, and other oral diseases using *in vitro* and *in vivo* models [9]. In addition, studies led by our group support its use as an antitumor agent for HNC, and in combating biofilms that contain disease-associated bacteria [7,8,14]. Specifically, we have shown nisin’s dose-dependent efficacy in abrogating the growth of pathogenic planktonic bacteria and bacteria present in oral biofilms associated with caries, periodontal disease, and persistent endodontic infections without inducing cytotoxicity to human oral cells [8]. Furthermore, we have shown nisin’s efficacy in abrogating HNC carcinogenesis and in extending survival in HNC mouse models [14]. However, a nisin-producing probiotic has not been tested for its effects on oral biofilms, especially those relevant to oral diseases.

Therefore, the objective of this current study was to test whether the nisin-producing probiotic *L. lactis* can promote oral healthy biofilms and prevent oral disease-associated biofilms.

## Material and methods

### Materials

NisinZ®P was purchased from Handary S.A. (Belgium). Nisin-producing *L. lactis* sp. *lactis* (Cat# 11454), *Treponemadenticola* (ATCC 35405), *Porphyromonas gingivalis* (ATCC 33277), and *Fusobacterium nucleatum* (ATCC 25586) were purchased from ATCC. The non-nisin producing *L. lactis* was a gift from Paul Cotter, from the Cork Institute of Technology. Brain Heart Infusion (BHI) media, a crystal violet stain, and all other general chemicals were purchased from Sigma-Aldrich (IL). The LIVE/DEAD BacLight Bacterial Viability Kit and Blocking buffer were purchased from ThermoFisher Scientific (USA). The 16S sequencing Explorer Kit was obtained from uBiome (USA).

### Nisin solution preparation

The Nisin Z (>95% purity) solution was prepared by gently mixing nisin powder in Mili-Q water at 5 mg/mL in a 15 mL tube covered by aluminum foil. This covered tube was then placed on a rotator and mixed for 4 h to completely solubilize Nisin. Finally, the solution was filtered using a 0.22 µm syringe filter.

### Human saliva collection and informed consent

Human saliva collection was approved by the University of California San Francisco Institutional Review Board (IRB #17-21912, Reference #186994, approved on April 25^th^, 2017). The collection protocol was previously published by our group [8]. Briefly, ten healthy volunteers with no known health issues verbally consented to donate saliva for this study. No information from the volunteers was collected at any time prior to or at the time of saliva donation. Prior to the collection, the volunteers were informed not to eat, drink and/or smoke for, at least, 30 min before the donation. They were comfortably seated and given a sterile tube for saliva collection. About 10–15 mL of saliva was obtained from each volunteer. All the collected saliva was pooled, centrifugated (10,000 x RPM for 30 min) and separated into a Cell-Containing Saliva (CCS) and Cell-Free Saliva (CFS). CCS was used as the biofilm inoculum and it was obtained by adding glycerol (50% v/v) to the precipitate of the centrifuged pooled saliva and stored at −80°C. CFS was used as biofilm medium and it was obtained by collecting supernatant of the centrifuged pooled saliva, diluted with sterile Phosphate Buffer Saline (PBS) (1:4 v/v) and stored at −80°C.

### Bacteria and biofilm growth

*T. denticola, P. gingivalis*, and *F. nucleatum* were grown as described previously [15–17]. *T. denticola* was cultured in Oral Treponeme Enrichment Broth (OTEB), while *P. gingivalis* and *F. nucleatum* were cultured in Brain-Heart Infusion (BHI) broth supplemented with hemin (5 µg ml^−1^) and vitamin K (1 µg ml^−1^) under anaerobic conditions. Anaerobic conditions were obtained by placing bacterial samples into sealed anaerobic jars that underwent five cycles of depressurization (vacuum formation) and Nitrogen (N_2_) pressurization (1 ATM) and kept at 37°C in a Fisher-Scientific Isotemp Incubator. The bacteria were split every 4–7 days. Purity of the spirochete cultures was confirmed by dark field microscopy, while the other two bacteria were confirmed by microscopic evaluation, colony morphology, and sequencing prior to use in experiments.

Both nisin-producing and non-nisin-producing *L. lactis* strains were grown in BHI overnight at 37°C with shaking and under aerobic conditions in an Eppendorf G24 Environmental Incubator Shaker.

The human saliva-derived oral biofilms were grown by adding 15 µL of CCS to 485 µL of CSF per well in 24-well plates and incubated under aerobic conditions for 24 h or 48 h at 37°C in a humidified Thermo-Fisher Forma Series II Incubator. CFS medium was changed every 24 h. For the pathogenic-spiked biofilms, 48 h preformed biofilms were spiked with 6 × 10^5^ CFU/mL of each periodontal pathogen (*T. denticola, F. nucleatum*, and *P. gingivalis)* and incubated under aerobic conditions for another 24 h.

Previously, others have determined that 0.1 OD_600_ is equivalent to 2.4 × 10^8^ CFU/mL of *T. denticola* [15] or *P. gingivalis* [18,19], 1 × 10^8^ CFU/mL of *F. nucleatum* [20] and to 6 × 10^7^ CFU/mL of *L. lactis* [21].

### Oral biofilm growth prevention

Oral biofilm growth prevention was measured using crystal violet staining or fluorescence confocal microscopy.

For crystal violet measurements, increasing concentrations of either *L. lactis* strains or nisin were added together with the initial biofilm inoculum and incubated for 24 h. Biofilms were fixed by heating and then biofilm biomass was quantified using crystal violet staining, as previously described [22,23]. Plates containing biofilms were washed with Milli-Q water and incubated at 50°C for 90 min. Then, 200 µL of crystal violet solution (0.06%) was added to each well and incubated for 10 min at room temperature (RT). The excess stain was removed by washing three times. Then, 300 µL of acetic acid was added to each well and incubated for 20 min at RT. The supernatant was then collected, equally divided into three 96-well plate wells and quantified by optical density (600 nm) in a Spectramax M2 microplate reader (Molecular Devices, USA).

For confocal microscopy, biofilms co-inoculated with either *L. lactis* strains or nisin were grown on 13 mm sterile coverslips for 24 h. Then, the biofilm was fixed with 4% paraformaldehyde for 5 min at RT, washed three times with PBS and stained using the LIVE/DEAD™ BacLight™ Bacterial Viability Kit following the manufacturer’s instructions. This kit is composed of two fluorophores: Syto9 and propidium iodide. Syto9 is a permeable green-fluorophore, thus, staining both live and dead cells as green. Propidium iodide in an impermeable red-fluorophore and it has 2-fold more affinity for nucleic acids than Syto9. Thus, propidium iodide displaces Syto9 and only stains dead cells as red [24]. Briefly, 5 µL of Syto9 and propidium Iodide stains were mixed together in 10 mL of PBS and incubated for 15 min at RT in the dark. The coverslips were washed three times, mounted, analyzed by using a TCS SP8 Confocal Microscope (Leica, Germany) and quantified using Fiji software [25].

### Oral biofilm disruption

Oral biofilm disruption was measured using crystal violet staining or fluorescence confocal microscopy.

For the crystal violet measurements, oral biofilms were pre-grown for 24 or 48 h. If the biofilms were spiked, the periodontal pathogens were added to the biofilms and incubated for another 24 h. Then, the spiked- or non-spiked biofilms were challenged with either *L. lactis* strains or nisin and incubated for another 24 h. Biofilms were then, fixed and the biofilm biomass was quantified using crystal violet, as described in the growth prevention assay.

For confocal microscopy, oral biofilms were pre-grown for 24 or 48 h on 13 mm sterile coverslips. If the biofilms were spiked, the periodontal pathogens were added to the biofilm and incubated for another 24 h. Then, the spiked- or non-spiked biofilms were challenged with either *L. lactis* strains or nisin and incubated for another 24 h. Finally, the biofilms were fixed, mounted, and analyzed as described in the growth prevention assay.

### 16S rRNA sequencing

All biofilms were collected using sterilized cotton swabs and mixed with the buffer supplied with the Ubiome’s Explorer kit (uBiome, USA) and placed in the provided shipping tubes. These tubes were sealed and sent to uBiome (USA) for 16S rRNA sequencing. uBiome sequenced the samples in a NextSeq 500 Series System (Illumina, USA). After the sequencing, uBiome supplied the resultant fastq files with Q-score > 30 (99.9% accuracy) in a paired-end modality.

The methodology used for processing the raw data was the following. Primers and any leading random nucleotides were trimmed out from the sequences. Forward reads were capped at 125bp and reverse reads were capped at 124bp. Sequences that contained more than eight of the same consecutive nucleotides (repeats) were also discarded. Next, the remaining sequences were clustered using a distance of one nucleotide using the Swarm algorithm [26], and the most abundant sequence per cluster was considered the representative of the cluster. Subsequently, a chimera removal algorithm (i.e. VSEARCH uchime_denovo [27]) was used on these centroid representative sequences and any singletons that remained after chimera removal were discarded. Finally, both forward and reverse reads that matched with at least 95% sequence identity to the same sequence in version 123 of the SILVA ribosomal RNA gene database [28] were assumed to be 16S sequences.

The taxonomic annotation was assigned according to the following similarity thresholds: 1) Sequences whose hits had >95% sequence identity were annotated to the same genus of the hit in the SILVA database; and 2) only hits with >97% sequence identity were annotated as same species of the hit in the SILVA database. The final analysis yielded more than 100,000 reads per sample. Then, RStudio software (RStudio, USA) was used to evaluate richness and diversity through the Shannon diversity index.

### T. denticola and P. gingivalis detection in oral biofilms by qPCR

Forty-eight-hour pre-grown oral biofilms were spiked with pathogens as described above. Then, the samples were collected and resuspended in PBS containing 20 mg/ml of lysozyme and were incubated for 0.5 h at RT to lyse the bacterial cell wall. Subsequently, DNA was extracted from the biofilms using the QIAamp DNA mini kit (Qiagen, Hilden, Germany) according to manufacturer’s instructions for DNA purification from Body Fluids using their spin protocol. After DNA purification, the ammount of total DNA was quantified from triplicate samples using a Nanodrop^TM^ One Microvolume UV-Vis Spectrophotometer (ThermoFisher Scientific, USA). Then, 1.2 µL of each sample in duplicates were subjected to 50 cycles of quantitative PCR containing the primers below for either *T. denticola* or *P. gingivalis* and using a QuantStudio 3 qPCR system.

**Table:** 

PCR primers	
Bacterium	Primer sequence (5ʹ-3ʹ)
*P.gingivalis*	F: AGT CGA GTT GCA GAC TCC GAT CC
	R: AAC CCA CAT CGG TAG TTG CTA ACA G
*T.denticola*	F: AGGGATATGGCAGCGTAGCA
	R: TTGCGGGACTTAACCCAACA

### Data availability

The datasets generated and/or analyzed during the current study are available from the corresponding author on reasonable request.

## Results

### Nisin-producing probiotic inhibits oral biofilm formation, structure, and viability

We previously showed that nisin inhibits and disrupts oral biofilm formation [8]. Here we explored the potential for the nisin-producing probiotic, namely *L. lactis* sp. *lactis* to modify oral biofilm properties. To analyze the probiotic effects on oral biofilms, an *in vitro* oral biofilm model system, which is based on biofilms formed from human saliva collected from healthy individuals, was established (Figure S1).

**Figure 1.**
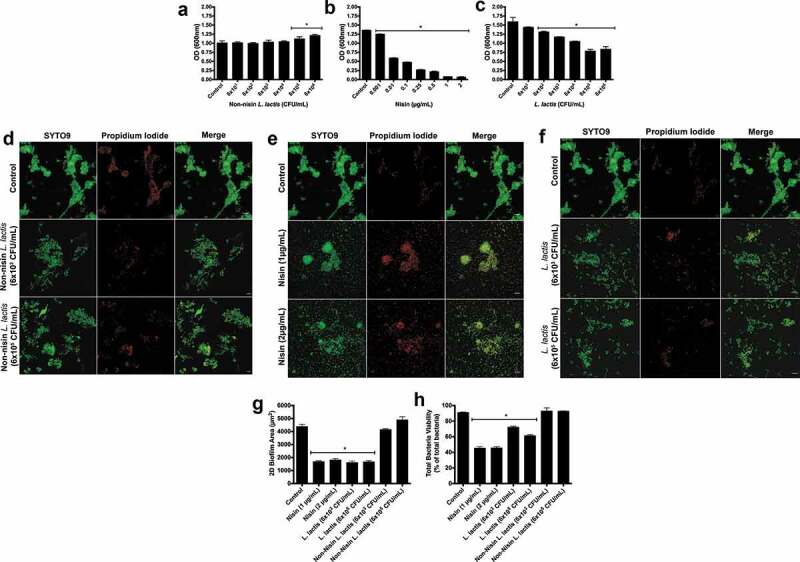
**Nisin-producing probiotic inhibits oral biofilm formation, structure, and viability.**

Initially, to examine the ability of the probiotic to prevent biofilm growth, we co-inoculated the biofilms with the non-nisin producing *L. lactis* (negative control), the purified nisin (positive control), or the probiotic (nisin-producing *L. lactis*) with the saliva-derived inoculum, then analyzed biofilm biomass using crystal violet staining (Figure 1a-c, respectively). The nisin-producing *L. lactis* significantly inhibited biofilm formation in a colony-forming unit (CFU)-dependent manner from 6 × 10^2^ to 10^6^ CFU/mL, whereas the non-nisin producing *L. lactis* (negative control) did not inhibit biofilm formation. In fact, at high concentrations (6 x 10^5−6^CFU/ml) the negative control even increased biofilm biomass. As expected from our previously published studies, the purified nisin bacteriocin (positive control) inhibited biofilm formation in a dose-dependent manner from 0.001 to 2 µg/ml. These data indicate that nisin is a significant effector molecule in biofilm growth inhibition, whereas bacterial competition via *L. lactis* has no/minimal contribution to the inhibition effects.

Biofilms were grown with non-nisin producing *L. lactis* (panels a and d), nisin (panels b and e) and nisin producing *L. lactis* (panels c and f) for 24 h. Changes in biofilm biomass were then measured as the optical density of the crystal violet staining at 600 nm; *means statistical difference (p < 0.05) between the marked sample and control. Panels d, e, and f show representative images of fluorescently labeled biofilms under different treatment conditions. The columns, from left to right, represent the different staining conditions; SYTO9, a membrane permeable and live cell stain and propidium iodide, a membrane-impermeable dead-cell stain, and the merged image shows the double and overlapping staining pattern (Syto 9 and propidium iodide). The rows represent the different treatments applied; from top to bottom, no treatment (Control), 6 × 10^3^ and 10^5^ CFU/mL of non-nisin producing *L. lactis*, 1 and 2 µg/mL of nisin treatment; 6 × 10^3^ and 10^5^ CFU/mL of nisin-producing *L. lactis*. Scale bar represents 10 µm. Quantification of Syto9 and PI labelled bacteria from confocal images. *means statistical difference (p < 0.05) between the sample and control. The 2D biofilm area (g) and viability (h) were quantified for treatment groups represented in panels d, e, and f.

To further examine the effects of nisin and the nisin-producing *L. lactis* on biofilm inhibition properties, we selected two concentrations of nisin (1 and 2 µg/mL) and *L. lactis* (6x10^3^ and 6 × 10^5^ CFU/mL) to explore their effects on oral biofilm structure and viability using immunofluorescence. Representative images can be seen in Figure 1(d-f) of biofilms grown after inhibition with the non-nisin producing *L. lactis*, nisin, and nisin-producing *L. lactis*. FIJI software was then used to quantify the resultant 2D biofilm area and viability, and the results can be seen in Figure 1(g,h). The probiotic (nisin-producing *L. lactis*) significantly decreased the 2D biofilm area in a CFU-independent manner and cell viability in a CFU-dependent manner. The positive control (nisin) also significantly decreased both biofilm parameters in a dose-independent manner. However, no statistical difference was found for the negative control (non-nisin producing *L. lactis*). Corroborating the biomass inhibition data, nisin plays a critical role in inhibiting the initial biofilm structure and overall cell viability during biofilm growth. However, in the absence of nisin production, the *L. lactis* bacterium does not seem to contribute to these effects.

### Nisin-producing probiotic disrupts oral biofilm formation, structure, and viability

**Figure 2.**
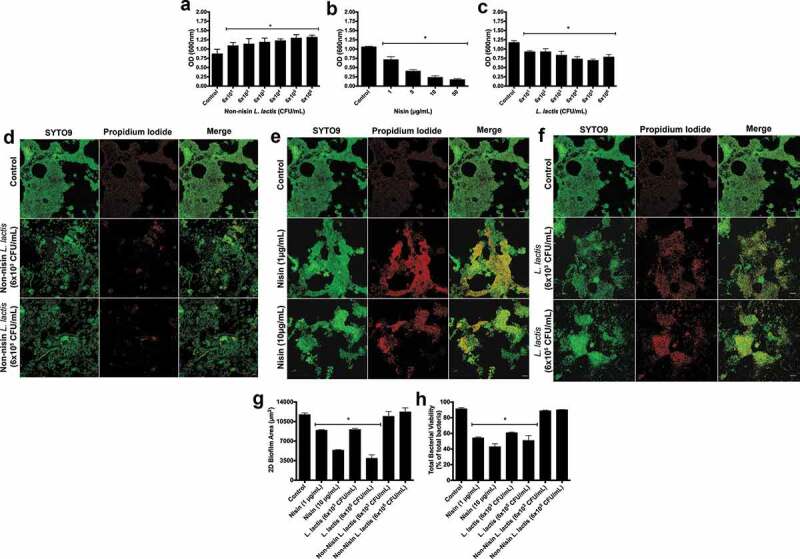
**Nisin-producing probiotic disrupts oral biofilm formation, structure, and viability.**

To further examine the probiotic’s ability to alter other biofilm parameters, we explored its ability to disrupt established or preformed biofilms. To this end, we preformed biofilms for 24h (Figure S2) and 48 h (Figure 2), then analyzed the ability of the nisin probiotic to disrupt biofilm biomass by inoculating established biofilms with the non-nisin producing *L. lactis* (negative control), the purified nisin (positive control), or the probiotic (nisin-producing *L. lactis*) (Figure 2(a-c), respectively). Similar to the biofilm inhibition or prevention, the probiotic and nisin significantly disrupted biofilm biomass in a dose- or CFU-dependent manner. In contrast, the negative control did not disrupt biofilm biomass, but even promoted biofilm biomass in a CFU-dependent manner from 6 × 10^1^ to 10^6^ CFU/mL. These data indicate that nisin effectively and dose-dependently disrupts biofilm biomass, whereas the *L. lactis* bacterium actively promotes the growth of established biofilms.

Biofilms were grown for 48 h, then treated for 24 h with non-nisin producing *L. lactis* (a and d), nisin (b and e), and nisin-producing *L. lactis* (c and f), then changes in biofilm biomass (a-c), 2D biofilm area (g), and viability (h) were evaluated. For the biofilm biomass, the results were measured as the optical density of the crystal violet staining at 600 nm. *means statistical difference (p < 0.05) between the marked sample and control. Panels d, e, and f show representative images of fluorescently labeled biofilms under different treatment conditions. The columns, from left to right, represent the different staining conditions; SYTO9, a membrane permeable and live cell stain and propidium Iodide, a membrane-impermeable dead-cell stain, and the merged image show the double and overlapping staining pattern (Syto 9 and propidium iodide). The rows represent the different treatments applied; from top to bottom, no treatment (Control), 6 × 10^3^ and 10^5^ CFU/mL of non-nisin producing *L. lactis*, 1 and 10 µg/mL of nisin treatment; 6 × 10^3^ and 10^5^ CFU/mL of nisin-producing *L. lactis*. Scale bar represents 10 µm. Quantification of Syto9 and PI labelled bacteria from confocal images. *means statistical difference (p < 0.05) between the sample and control. The 2D biofilm area (g) and viability (h) were quantified for treatment groups represented in panels d, e, and f.

We further analyzed the probiotic’s effect on the structure (Figure 2(d-g)) and viability (2d-f, h) of preformed biofilms using immunofluorescence. Similar to biomass, *L. lactis* and nisin significantly disrupted preformed biofilm structure and viability, although in a concentration and CFU-dependent manner. In contrast, the negative control did not significantly disrupt the 2D biofilm structure or cell viability.

### Nisin-producing probiotic disrupts pathogen-spiked oral biofilm formation, structure, and viability

**Figure 3.**
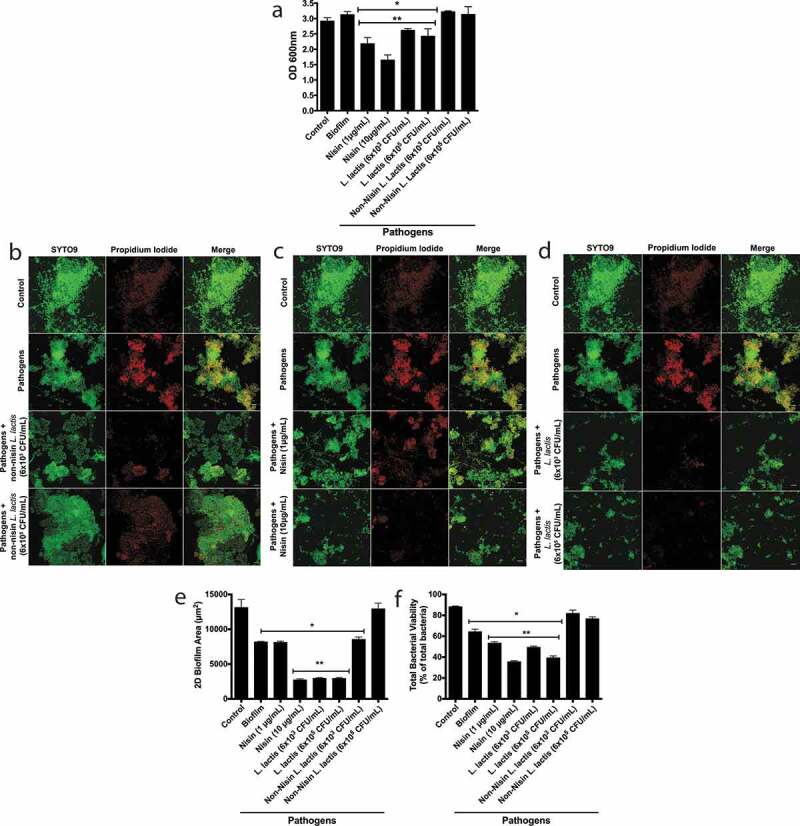
**Nisin-producing probiotic disrupts pathogen-spiked oral biofilm formation, structure, and viability.**

To examine the ability of the *L. lactis* probiotic to alter pathogen-spiked biofilm properties, we infected 48 h preformed biofilms with *P. gingivalis, F. nucleatum* and *T. denticola*, then incubated the biofilms for an additional 24 h. We next treated the pathogen-spiked biofilms with two concentrations of the non-nisin producing *L. lactis* (negative control), nisin (positive control), and the nisin-producing *L. lactis*. The resulting biofilms were analyzed for biofilm biomass (Figure 3(a)). Both the probiotic and nisin significantly disrupted the pathogen-spiked biofilm biomass. In contrast, the non-nisin producing *L. lactis* (negative control) was not able to disrupt the pathogen-spiked biofilm biomass. We further analyzed the biofilm structure (Figure 3(b-d)) and quantified the 2D biofilm area (Figure 3(e)) and cell viability (Figure 3(f)). Although there was no significant difference between the control and pathogen-spiked biofilm biomass, the 2D biofilm area and cell viability were significantly different. This demonstrates that the pathogens significantly changed the overall biofilm structure and viability compared to control biofilms. Specifically, spiking the biofilms with pathogens reduced the biofilm 2D area by 40% and total viability by 23%. The probiotic (nisin-producing *L. lactis*) and nisin were able to significantly reduce both the 2D area and viability of the pathogen-spiked biofilms, whereas the non-nisin producing *L. lactis* (negative control) was not able to disrupt the pathogen-spiked biofilm 2D area and viability.

Biofilms were grown for 48 h, then spiked with *P. gingivalis, T. denticola*, and *F. nucelatum* for 24 h, and finally treated for 24 h with non-nisin producing *L. lactis* (a,b), nisin (a,c), and nisin-producing *L. lactis* (a,d), then changes in biofilm biomass (a), 2D biofilm area (b-d,e), and viability (b-d,f) were evaluated. For the biofilm biomass, the results were measured as the optical density of the crystal violet staining at 600 nm. *means statistical difference (p < 0.05) between the marked sample and control. Panels b, c, and d show representative images of fluorescently labeled biofilms under different treatment conditions. The columns, from left to right, represent the different staining conditions; SYTO9, a membrane permeable and live cell stain and propidium Iodide, a membrane-impermeable dead-cell stain, and the merged image show the double and overlapping staining pattern (Syto 9 and propidium iodide). The rows represent the different treatments applied; from top to bottom, no treatment (Control), pathogen infected, pathogen infected and treated with 6 × 10^3^ and 10^5^ CFU/mL of non-nisin producing *L. lactis*, pathogen infected and treated with1 and 10 µg/mL of nisin treatment; and pathogen infected and treated with 6 × 10^3^ and 10^5^ CFU/mL of nisin-producing *L. lactis*. Scale bar represents 10 µm. Quantification of Syto9 and PI labelled bacteria from confocal images. *means statistical difference (p < 0.05) between the sample and control biofilms. **mean statistical difference (p < 0.05) between the sample and the pathogen-spiked biofilms (Biofilm). The 2D biofilm area (e) and viability (f) were quantified for treatment groups represented in panels b, c, and d.

### Biofilm bacterial diversity returns to control levels with nisin-producing probiotic or nisin

Next, we sequenced the 16S rRNA of the oral microbiome of the control and pathogen-spiked biofilms in order to examine changes in biofilm community composition mediated by the probiotic treatment. One of the main techniques for examining differences in a microbial community (e.g. oral microbiome) is using diversity indexes [29,30]. Diversity indexes provide information about the total number of species in a community (i.e. richness), but also about the rarity and commonness of each species within that community. Thus, this tool is very important for understanding community composition. Among the diversity indexes, the Shannon Index (H) is one of the most commonly used indexes for characterizing microbial diversity [31]. This Index accounts for both abundance and rarity of the species present in the community by multiplying the proportion of a species *i* found (p_i_) by the natural logarithm (*ln*) of this proportion. The resulting product is, then, summed across all species (*s*), which correlates to the richness of the community and multiplies it by −1, as described by equation below [32].

$$H=-\overset{s}{\underset{i=1}{\sum }}p_{i}ln\left(p_{i}\right)$$

**Figure 4.**
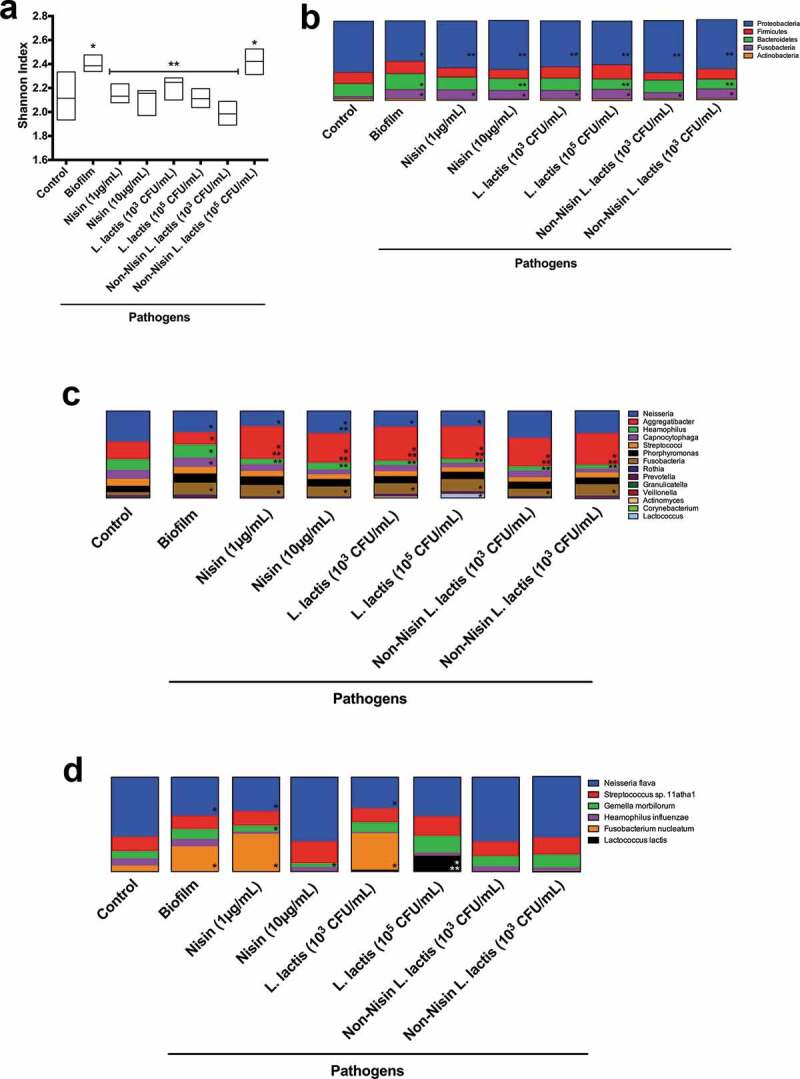
**Biofilm bacterial diversity returns to control levels with nisin-producing probiotic or nisin.**

Using the Shannon diversity index, we analyzed the ecological effects of the non-nisin-producing *L. lactis* (negative control), the nisin (positive control) and the probiotic (nisin-producing *L. lactis*) on the pathogen-spiked oral biofilms (Figure 4(a)).

Biofilm Shannon (a) Diversity Index and Phyla (b), Genera (c) and Species (d) community composition are illustrated, after treatment with nisin, nisin-producing *L. lactis* and non-nisin producing *L. lactis* in the pathogen-spiked 48 h preformed biofilms, with data obtained from 16S rRNA sequencing analysis. * represents significant difference (p < 0.05) between sample and non-pathogenic biofilms (Control). ** represent significant difference (p < 0.05) between sample and the pathogen spiked biofilms (Biofilm).

The pathogen-spiked biofilms had a higher level of diversity compared to the control biofilms. All nisin-based treatments, including the probiotic (nisin-producing *L. lactis*) and the nisin (positive control) significantly returned the diversity index back to control levels, except for the negative control (non-nisin producing *L. lactis*) at 6x10^5^ CFU/mL. This demonstrates that nisin promotes changes in the biofilm community composition by potentially targeting specific bacterial species. Further, the non-nisin producing *L. lactis* at higher CFUs can effectively change the bacterial diversity back to control levels, despite not disrupting the biofilm structure.

### Nisin-producing probiotic and nisin significantly suppress F. nucleatum, T. forsythia, P. gingivalis, T. denticola, and Klebsiella pneumoniae, while promoting commensals in pathogen-spiked oral biofilms

The effects of the probiotic (nisin-producing *L. lactis*), the non-nisin-producing *L. lactis*, and nisin on the microbiome of the control and pathogen-spiked biofilms was also evaluated by phyla, genera, and species composition (Figure 4(b-d), respectively). The phyla analysis demonstrates that all treatments return the *Proteobacteria* back to control levels. Interestingly both *L. lactis* strains and nisin at their highest tested concentrations (10 µg/mL or 10^5^ CFU/mL) but not at their lower concentrations (1 µg/mL and 10^3^ CFU/mL) returned *Bacteroidetes* taxa back to control levels. However, the *Fusobacteria* taxa levels in the pathogen-spiked biofilms did not seem to be affected by any of the treatments.

The genera analysis demonstrated that the nisin probiotic and nisin significantly affected *Neisseria, Haemophillus, Aggregatibacter*, and *Lactobacillus* in pathogen-spiked biofilms. Specifically, the *Haemophillus* genus was suppressed, whereas *Aggregatibacter* and *Lactobacillus* were elevated beyond control levels. Intriguingly, the *Neisseria* genus returned to control levels after either nisin (10 µg/mL) and non-nisin producing *L. lactis* treatment, but not after treatment with nisin-producing *L. lactis*. The combined effects of nisin and *L. lactis* may present a significant competition that is detrimental for the survival of the *Neisseria* genus.

**Figure 5.**
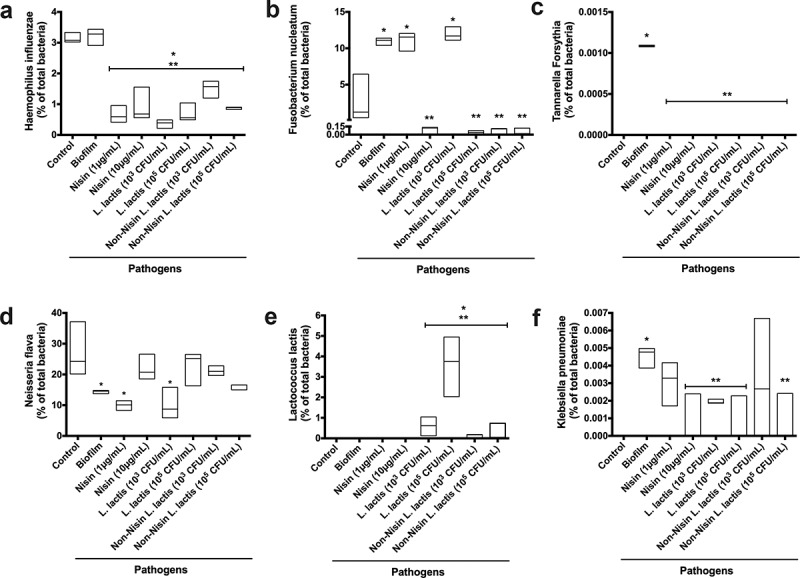
**Nisin-producing probiotic and nisin significantly suppress** ***Fnucleatum, T.forsythia*** **and** ***K.pneumoniae*****, while promoting commensals in pathogen-spiked oral biofilms.**

Further analysis of the 16S rRNA sequencing data at the species level (Figures 4(d) and 5) demonstrated that the nisin-producing probiotic or nisin significantly suppressed *Haemophilus influenzae, F. nucleatum, Tannarella forsythia*, and *Klebisiella pneumoniae* pathogenic species, while promoting the commensal *Neisseria flava*, and *L. lactis* (Figure 5(a-f)) species levels in pathogen-spiked biofilms.

The levels of *H. influenzae* (a), *F. nucleatum* (b), *T. forsythia* (c), *N. flava* (d), *L. lactis* (e), and *K. pneumoniae* (f) obtained from 16S rRNA sequencing analysis in the pathogen-spiked biofilms after treatment with nisin, nisin-producing *L. lactis* and non-nisin producing *L. lactis* are illustrated in the graphs as a percentage of the total bacteria present in the biofilms. * indicates significant difference (p < 0.05) between the sample genera and control genera. ** represent significant difference (p < 0.05) between the sample genera and biofilm (pathogen containing) genera.

Although not present in the 16S rRNA sequencing, presence of *T. denticola* and *P. gingivalis* were confirmed in the pathogen-spiked biofilm by PCR.

## Discussion

Oral biofilms play an essential role both in the development of natural oral physiology and defense of the host [5]. Because of these important roles, an imbalance in the oral microbiome or dysbiosis is associated with a variety of oral and systemic diseases, including periodontal disease, caries, recurrent endodontic infections, and HNC [6,7]. Although antibiotics can be used to address this dysbiosis, they have limitations, including the incidence of bacterial resistance and concerns over wide-spread environmental use. Thus, new strategies to modulate oral biofilm dysbiosis, especially those associated with disease are needed. Therefore, the objective of this current study was to test whether the nisin-producing *L. lactis* probiotic can promote healthy oral biofilms and prevent oral disease-associated biofilms.

The oral biofilms in this study were grown in aerobic conditions to replicate the overall conditions in the oral cavity, which has different levels of aerobic and anaerobic conditions. The oral biofilm itself experiences different levels of oxygenation. The biofilm surface is considered aerobic (>0.5% of oxygen), whilst the inner regions are considered anaerobic (<0.5% of oxygen) [33]. Thus, in this context, the biofilm itself contains the optimal conditions for the growth of different microbes that thrive under different levels of oxygenation. Thus, anaerobic compartments are present within the biofilm, where the oral pathogenic bacteria can grow. Furthermore, periodontal disease-associated pathogenic bacteria are oxygen-tolerant. For instance, *T. denticola* and *P. gingivalis* are considered facultative anaerobes [34,35], while *F. nucleatum* can grow under atmospheric conditions with more than 6% oxygen [36]. Thus, imposing absolute anaerobic growth conditions on the biofilm would not favor the growth of a complex biofilm community, including the largely aerobic commensal bacteria that seed the first layers of the biofilm, which are then followed by the pathogenic microbes that thrive within a spectrum of different levels of oxygenation [37].

Recently, our workgroup demonstrated that nisin can inhibit oral biofilm formation and disrupt pre-formed biofilms in a dose-dependent manner, without adversely affecting human oral cells [8]. We further showed that, in large part, pathogens were more sensitive to nisin compared to commensals, making the use of nisin a potential strategy for promoting ‘health’ in the oral microbiome, especially one affected by pathogenic bacteria. Therefore, the objective of this investigation was to test whether the nisin-producing probiotic *L. lactis* or its bacteriocin nisin could promote a healthier oral biofilm in the context of a pathogen spiked biofilm environment. However, the definition for oral biofilm ‘health’ is complex. First, it must be appreciated that there is a symbiotic relationship between the host (humans) and the commensal guests, which comprise the bacterial biofilms. In this context, the definition of health should include the benefit of both organisms (the host and the commensal guests). Second, it is difficult to define a normal or healthy microbiota because each individual is different, and given the high prevalence of dental caries and periodontal diseases [4], many of these pathogenic bacteria are present even in healthy individuals/sites, but at lower frequency than in the diseased individuals/sites [38]. Thus, taking these points into consideration, there are two approaches that can be used to analyze oral biofilm health.

The first approach is to analyze the shift in bacterial composition in oral biofilms. Recent literature has proposed that there is a distinct shift in bacterial composition when oral diseases are present [4,7,38]. In this context and in systemic diseases, several species have been identified as pathogenic, including *F. nucleatum* [39–41], *T. forsythia* [42–44], *H. influenzae* [45], and *K. pneumoniae* [46], such that high levels of these bacteria are associated with a different disease status [7,47,48]. Our data indicate that a nisin probiotic and/or nisin can reduce the levels of these pathogens down to control levels, while promoting commensal bacteria, such as *N. flava* [49]. Thus, in the broad context of the literature, nisin-containing treatments promote a shift of pathogenic biofilms towards healthier biofilms.

A second approach to understanding biofilm health is to consider the ecological levels of bacteria. Unlike other ecosystems (e.g. gut microbiome), oral health is typically associated with low diversity and richness, whereas oral diseases are associated with an increasing oral microbiome richness and diversity [6]. Regarding this concept of oral biofilm health, our data indicate that treatment with the probiotic *L. lactis* and its bacteriocin can shift the diversity of the oral biofilm towards control levels. Thus, this indicates a shift towards health in the pathogen-spiked biofilms.

## Conclusion

We conclude that the nisin-producing probiotic *L. lactis* and its purified bacteriocin, namely, nisin, can prevent and disrupt oral biofilms, decrease the amount of oral pathogens within oral biofilms, and return the diversity of oral biofilms to control levels. Therefore, both of these agents may be useful to promote healthier oral biofilms and, in turn, improve a patient’s oral health.

## Supplementary Material

Supplemental Material
