# Supplementary material for: Modulation of pathogenic oral biofilms towards health with nisin probiotic
Source: J Oral Microbiol. 2020 Aug 24;12(1):1809302. doi: 10.1080/20002297.2020.1809302 (PMC7482728; doi:10.1080/20002297.2020.1809302)
Supplement: Supplemental Material [file ZJOM_A_1809302_SM6634.zip › Supplementary/Figure 1.pdf]

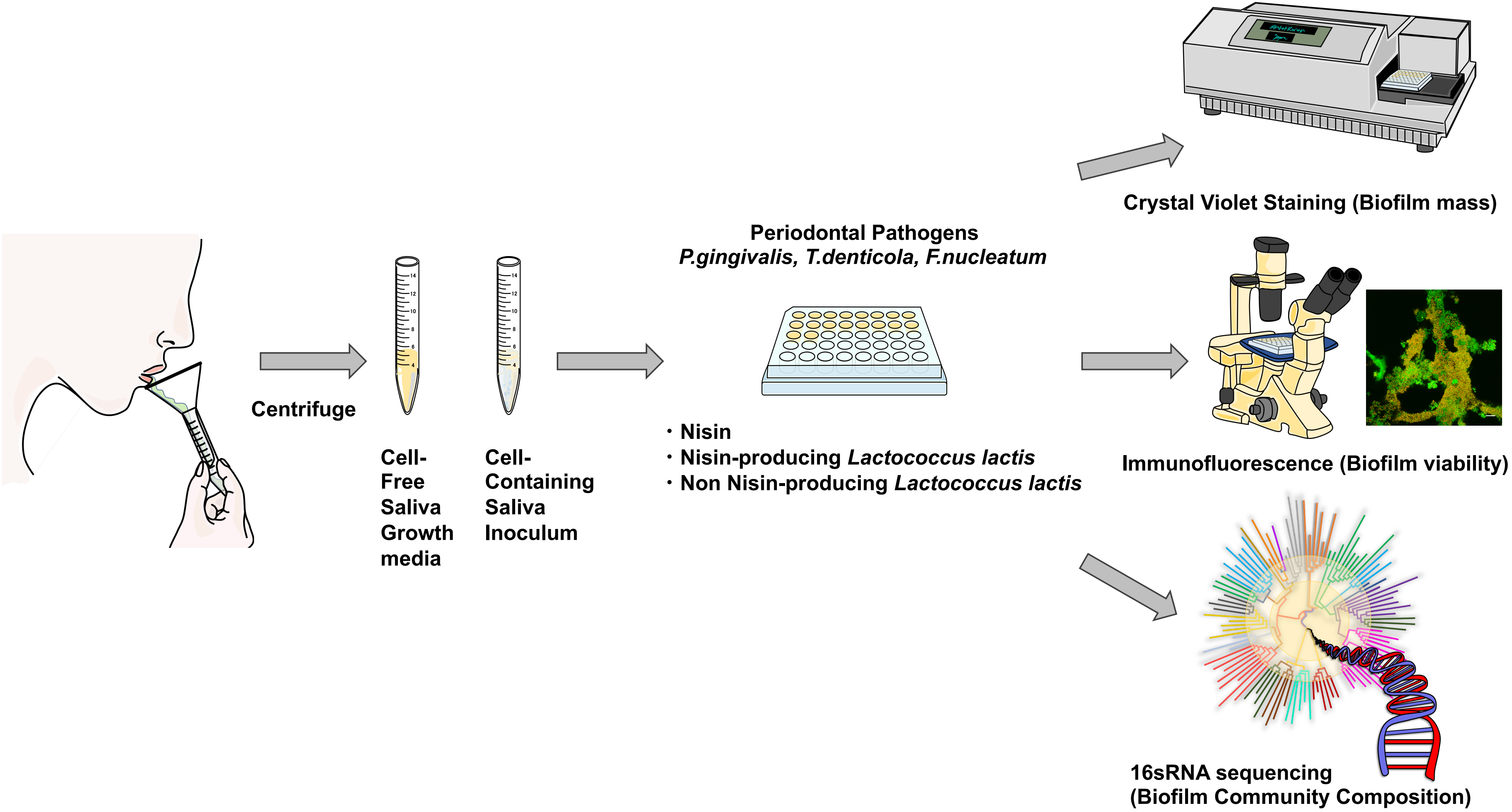

Crystal Violet Staining (Biofilm mass)
Periodontal Pathogens
P.gingivalis, T.denticola, F.nucleatum
Centrifuge
Cell-Free Saliva Growth media
Cell-Containing Saliva Inoculum
・Nisin
・Nisin-producing Lactococcus lactis
・Non Nisin-producing Lactococcus lactis
Immunofluorescence (Biofilm viability)
16sRNA sequencing
(Biofilm Community Composition)
